# Inhibition of the prolyl isomerase Pin1 enhances the ability of sorafenib to induce cell death and inhibit tumor growth in hepatocellular carcinoma

**DOI:** 10.18632/oncotarget.15967

**Published:** 2017-03-07

**Authors:** Min Zheng, Huijuan Xu, Xin-Hua Liao, Champ Peng Chen, Arina Li Zhang, Wenxian Lu, Long Wang, Dayun Yang, Jichuang Wang, Hekun Liu, Xiao Zhen Zhou, Kun Ping Lu

**Affiliations:** ^1^ Fujian Key Laboratory for Translational Research in Cancer and Neurodegenerative Diseases, Institute for Translational Medicine, School of Basic Medical Sciences, Fujian Medical University, Fuzhou, Fujian 350108, China; ^2^ Key Laboratory of Ministry of Education for Gastrointestinal Cancer, Fujian Medical University, Fuzhou, Fujian 350108, China; ^3^ Division of Translational Therapeutics, Department of Medicine and Cancer Research Institute, Beth Israel Deaconess Medical Center, Harvard Medical School, Boston, Massachusetts 02215, USA

**Keywords:** sorafenib, Pin1, ATRA, HCC, cell death

## Abstract

Hepatocellular carcinoma (HCC) is the sixth most common cancer, but is the second leading cause of cancer deaths, partially due to its heterogeneity and drug resistance. Sorafenib is the only medical treatment with a proven efficacy against advanced HCC, but its overall clinical efficacy is still modest. Therefore, a major challenge is how to improve its therapeutic efficacy. The unique prolyl isomerase Pin1 regulates numerous cancer-driving pathways. Notably, Pin1 is overexpressed in about 70% HBV-positive HCC patients and contributes to HCC tumorigenesis. However, the role of Pin1 in the efficacy of sorafenib against HCC is unknown. Here we found that sorafenib down-regulated Pin1 mRNA and protein expression, likely through inhibition of Pin1 transcription by the Rb/E2F pathway. Importantly, Pin1 knockdown potently enhanced the ability of sorafenib to induce cell death in HCC, which was further supported by the findings that Pin1 knockdown led to stabilization of Fbxw7 and destabilization of Mcl-1. Furthermore, all-*trans* retinoic acid (ATRA), a known anticancer drug that inhibits and ultimately induces degradation of active Pin1 in cancer cells, also potently sensitized HCC cells to sorafenib-induced cell death at least in part through a caspase-dependent manner. Moreover, ATRA also synergistically enhanced the ability of sorafenib to reduce Pin1 and inhibit tumor growth of HCC in mouse xenograft models. Collectively, these results not only demonstrate that Pin1 down-regulation is a key event underlying the anti-tumor effects of sorafenib, but also uncover that Pin1 inhibitors offer a novel approach to enhance the therapeutic efficacy of sorafenib against HCC.

## INTRODUCTION

Liver cancer is the sixth most common cancer worldwide, with 782000 new cases diagnosed in 2012 [1]. In contrast to stable or declining trends of most cancers, the incidence rate of liver cancer increases from 2003 to 2012 in the United States [2]. More importantly, liver cancer has moved up to the second leading cause of cancer-related deaths in the world. This is partially due to lack of efficacious targeted therapy to control high inter- and intra-tumor heterogeneity and complexity of etiology in this cancer, with multiple cancer-driving pathways being often activated at the same time.

Sorafenib is the only medical treatment that was approved by the US Food and Drug Administration for the treatment of advanced hepatocellular carcinoma (HCC) with a proven efficacy [3–7]. Sorafenib was originally designed to target on the Raf family of protein kinases, which control cell proliferation and tumor development in many types of cancer including HCC [8]. Pharmacological profiling studies have identified several receptor tyrosine kinases as its targets, such as VEGFR1/2/3/, PDGFR, c-Kit and RET, conferring sorafenib as a multi-kinase inhibitor [9]. However, patients with advanced HCC only benefit no more than 3 months survival advantage from sorafenib treatment [4].

As a consequence of the lack of more effective compounds or treatment strategies, a major aim of biological research is to improve the efficacy of sorafenib against HCC [7]. VEGFA amplification was identified as a key biomarker for patients who might be sensitive to sorafenib treatment [10]. In another study, *in vivo* RNA interference screening targeting on genes located within focal genomic amplification identified MAPK14 as a key regulator of sorafenib resistance in liver cancer [11]. Combinational blockade of MAPK14 and other key regulators is proposed to overcome sorafenib resistance in human HCC [12]. These two pioneer works implicate a promise for sorafenib precision therapy and combinational therapy in HCC.

Recently, to enhance the ability of sorafenib to induce cell death in HCC has been proposed to be a new strategy. Sorafenib alone leads to apoptosis [13] or iron dependent cell death, named ferroptosis [14], in a cell type specific manner. The role of sorafenib in HCC cell death is attributed to down-regulating Bcl-2 family member, Mcl-1 (Myeloid Cell Leukemia-1) [15]. Sorafenib blocks Erk mediated Mcl-1 phosphorylation on Thr92, which de-stabilizes Mcl-1 [16]. On the other hand, sorafenib activates GSK3beta by attenuating the inhibitory phosphorylation on Ser9 [17]. Activated GSK3beta phosphorylates Mcl-1 on Ser159 and Thr163, leading to its interaction with Fbxw7, an E3 ubiquitin ligase [18]. Additional mechanisms have been reported in other cancer types. Sorafenib has been shown to perturb mitochondrial function and reduce intracellular ATP levels, leading to activation of AMP-activated protein kinase (AMPK) and inhibition of mTORC1 activity, which finally promotes cell death in breast cancer cells [19]. Sorafenib can also induce down-regulation of survivin, leading to apoptotic cell death in human non-small lung cancer cells [20]. However, Sorafenib does not target these proteins directly so that the upstream regulators for this process remain to be elucidated.

The unique prolyl isomerase, Pin1 is prevalently overexpressed or over-activated in many types of cancer including HCC [21, 22]. Accumulating evidences have demonstrated that Pin1 plays a key role in cancer development, progression and prognosis by turning on more than 40 oncogenes/growth-promoting proteins and turning off more than 20 tumor suppressors/growth-inhibiting proteins at the same time [21]. Pin1 catalyzes cis-trans isomerization of specific phosphorylated Ser/Thr-Pro motifs and induce conformational change of proteins after proline-directed Ser/Thr phosphorylation [23], thereby affecting activities and stabilities of its substrates [24]. Notably, Pin1 is specifically overexpressed in more than 70% HBV-related HCC in China [22, 25] and Pin1 overexpression transforms normal liver cells [26]. Interestingly, many mediators of sorafenib induced cell death, such as Fbxw7, Mcl-1, survivin and AMPK are phosphorylated on Ser/Thr-Pro motif and their protein stabilities and activities are regulated by Pin1-catalyzed cis-trans isomerization [16, 24, 27–29]. However, the role of Pin1 in the HCC treatment, especially sorafenib-based targeted therapy is still uncharacterized. Given the critic role of Pin1 in HCC development [30], we investigate whether Pin1 plays a role in anti-tumor effects of sorafenib in HCC. In this present study, we showed that Pin1 expression is down regulated upon sorafenib treatment and inhibition of Pin1 either by genetic or chemical ablation potentiates anti-tumor efficacy of sorafenib against HCC *in vitro* and *in vivo*.

## RESULTS

### Sorafenib down-regulates Pin1 expression in multiple human HCC cells

**Figure 1:**
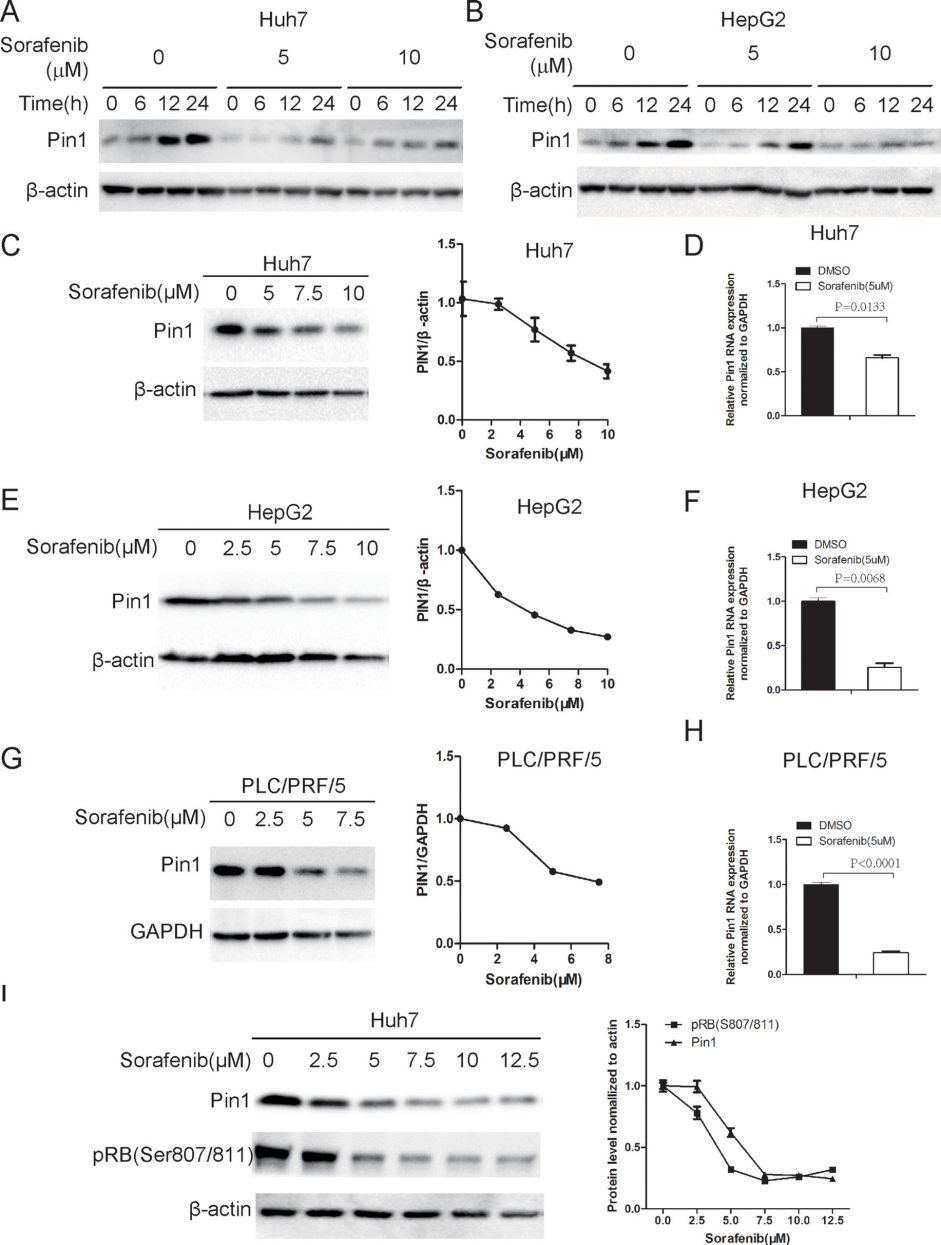
Sorafenib down-regulates Pin1 mRNA and protein expression (**A**, **B**) Sorafenib inhibited Pin1 biosynthesis and accumulation in Huh7 and HepG2 cells. Cells were treated with 5 or 10 μM sorafenib for indicated times. Pin1 protein expression was determined by Western Blot. (**C**, **E**, **G**) Sorafenib down-regulated Pin1 protein expression in multiple HCC cell lines. Huh7, HepG2 and PLC/PRF/5 cells were treated with different doses of sorafenib. Pin1 protein expression was determined by Western Blot. Expression of each protein was quantified using Image pro plus 6. (**D**, **F**, **H**) Sorafenib down-regulated Pin1 mRNA expression. Huh7, HepG2 and PLC/PRF/5 cells were treated with 5 μM sorafenib. Pin1 mRNA expression was determined by real-time PCR. (**I**) Down-regulation of RB phosphorylation on Ser807/Ser811 was associated with Pin1 protein expression in response to sorafenib treatment. Huh7 cells were treated with different doses of sorafenib for 24 hours. Phosphorylation of RB on Ser807/Ser811 and Pin1 expression were determined by Western Blot.

To determine the role of Pin1 in response of HCC cells to Sorafenib, we first examined the effect of sorafenib on Pin1 expression. Human HCC cell lines, Huh7 and HepG2 cells, were treated with sorafenib for indicated times. Sorafenib dramatically suppressed Pin1 biosynthesis and accumulation both in Huh7 and HepG2 cells (Figure 1A and 1B). Dose-response analysis for sorafenib induced Pin1 down-regulation was also performed in Huh7, HepG2 and PLC/PRF/5 cells. Sorafenib treatment led to a does-dependent decrease in Pin1 protein levels, as compared to vehicle controls in all HCC cell lines examined (Figure 1C, 1E and 1G). To further investigate whether sorafenib down-regulated Pin1 expression at the mRNA level, real-time PCR was performed to detect Pin1 mRNA levels in Huh7, HepG2 and PLC/PRF/5 treated with 5 μM sorafenib for 24 hours. Sorafenib treatments also significantly down-regulated Pin1 mRNA (Figure 1D, 1F and 1H). The ability of sorafenib to reduce Pin1 expression was further confirmed by the observations that sorafenib attenuated Rb phosphorylation on Serine 807/811 (Figure 1I) in a dose-dependent manner, which was correlated with down-regulation of Pin1 protein (Figure 1I). These results are consistent with the previous findings that the Rb-E2F pathway regulates Pin1 transcription [31] and that sorafenib inhibits the Rb-E2F pathway by reducing Rb phosphorylation on Serine 807/811 [32]. These data together show that sorafenib down-regulates Pin1 mRNA and protein expression in multiple human HCC cells.

### Pin1 knockdown potently enhances the ability of sorafenib to induce cell death

**Figure 2:**
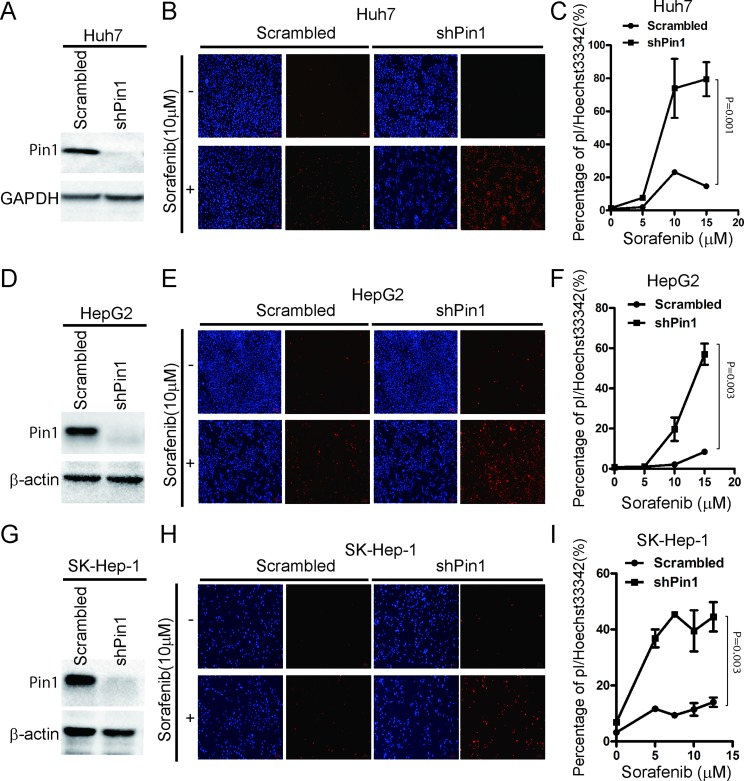
Knockdown of Pin1 sensitizes HCC cells to sorafenib-induced cell death (**A**, **D**, **G**) Pin1 was knocked down in multiple HCC cell lines. Huh7, HepG2 and SK-Hep-1 cells were knocked down using shRNA. Protein expression of Pin1 and beta-actin or GAPDH was determined by Western Blot. (**B**, **E**, **H**) Pin1 knockdown enhanced sorafenib induced cell death. Huh7, HepG2 and SK-Hep-1 cells were treated with 10 μM sorafenib. Cells were stained with pI and Hoechst 33342, photographed under microscopy. (Red: pI positive. Blue: Hoechst positive). (**C**, **F**, **I**) Pin1 enhanced cell death induced by sorafenib was dose dependent. Huh7, HepG2 and SK-Hep-1 cells were treated with different doses of sorafenib. Cells were stained with pI and Hoechst 33342. The number of red and blue spots were counted using Image pro plus 6. Sorafenib induced Cell death in Huh7, HepG2 and SK-Hep-1 cells were determined by the ratio of Red/Blue.

Inducing direct cytotoxicity in cancer cells is one of the main goals of anticancer treatments [33]. Although Sorafenib has been shown to induce cell death in HCC cells, it is weakly pro-apoptotic as a single agent [13]. In order to investigate the significance of Pin1 in sorafenib-induced cell death, we stably knocked down Pin1 expression in Huh7, HepG2 and SK-Hep-1 HCC cells using validated Pin1 shRNA lentiviruses, which led to effective Pin1 knockdown, as compared with scrambled shRNA control cells (Figure 2A, 2D and 2G), as described previously [34]. Pin1 knockdown and control cells were treated with 10 μM sorafenib for 72 hours and stained with propidium iodide (pI) and Hoechst33342, which have been previously shown to stain dead/late apoptotic and early apoptotic/normal cells, respectively [35, 36]. Silencing Pin1 expression by genetic knockdown drastically enhanced the ability of sorafenib to induce cell death in these HCC cell lines, as indicated by pI staining (Figure 2B, 2E and 2H). Moreover, the effects were also dependent on the dose of sorafenib (Figure 2C, 2F and 2I). These results demonstrate that Pin1 knockdown potently enhances the ability of sorafenib to induce cell death in multiple human HCC cells.

**Figure 3:**
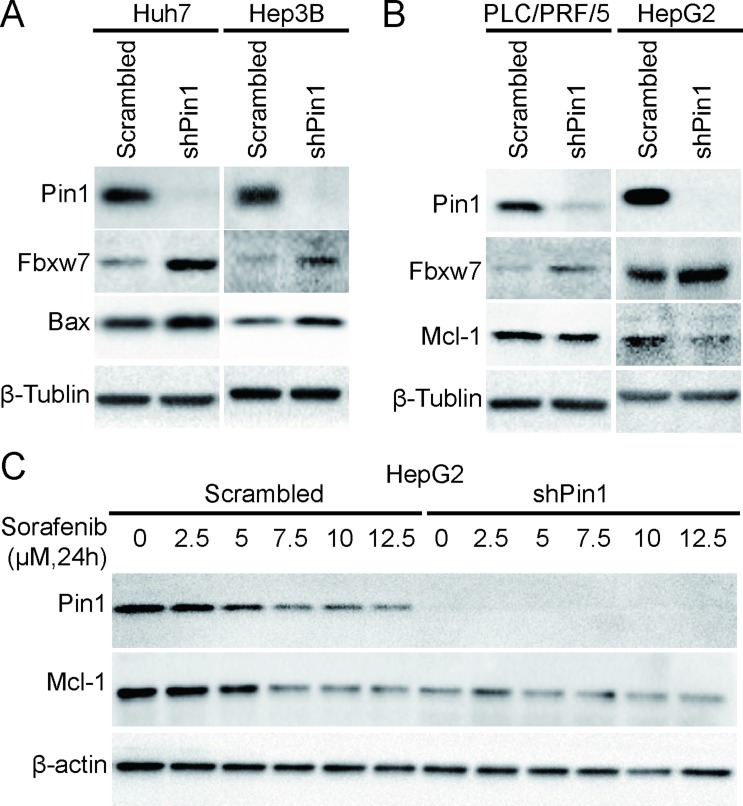
Pin1 affects sorafenib-induced cell death through regulation of Mcl-1 and Bax protein expression (**A**) Fbxw7 and Bax were up-regulated in Pin1 knockdown Huh7 and Hep3B cells. Pin1 was knocked down in Huh7 and Hep3B cells. Protein expression of Pin1, Fbxw7, Bax and beta-tubulin was determined by Western Blot. (**B**) Fbxw7 was up-regulated and Mcl-1 was down-regulated in Pin1 knockdown PLC/PRF/5 and HepG2 cells. Pin1 was knocked down in PLC/PRF/5 and HepG2 cells. Protein expression of Pin1, Fbxw7, Mcl-1 and beta-tubulin was determined by Western Blot. (**C**) Down-regulation of Mcl-1 is associated with Pin1 expression upon sorafenib treatment. Pin1 knocked down HepG2 and its counterpart cells were treated with different doses of sorafenib for 24 hours. Expression of Pin1, Mcl-1 and beta-actin was determined by Western Blot.

Next, we investigated the molecular mechanisms underlying the effects of Pin1 in sorafenib-induced cell death. Pin1 has been shown to regulate multiple anti- or pro-apoptotic proteins, such as Bax [37], Mcl-1 [16] and survivin [28]. Sorafenib has also been shown to induce cell death through activation of Bax [38] or down-regulation of Mcl-1 [39] and survivin [20]. The degradation of Mcl-1 has been shown to depend on Erk-mediated phosphorylation [16] and Fbxw7-mediated ubiquitin-proteasome pathway [18]. Interestingly, it has been shown that Pin1 stabilizes Mcl-1 through direct binding to Mcl-1 [16] and promoting Fbxw7 degradation [40]. Therefore, we examined the expression of these Pin1 substrate proteins in Pin1 knockdown and control cells. Pin1 knockdown led to up-regulation of Fbxw7 in Huh7, HepG2, Hep3B and PLC/PRF/5 cells (Figure 3A and 3B), as expected from the previous studies [40]. Bax activation was detected in Pin1 knockdown Huh7 and Hep3B cells (Figure 3A), while Mcl-1 down-regulation was detected in Pin1 knockdown PLC/PRF/5 and HepG2 cells (Figure 3B). Furthermore, sorafenib induced Mcl-1 degradation was dose-dependent and correlated with Pin1 down-regulation (Figure 3C). These data implicate that Pin1 shRNA potently enhances the ability of sorafenib to induce cell death in HCC cells, which is further supported by the findings that Pin1 knockdown leads to the stabilization of Fbxw7 and the destabilization of Mcl-1.

### The Pin1 chemical inhibitor ATRA sensitizes HCC cells to sorafenib-induced cell death

**Figure 4:**
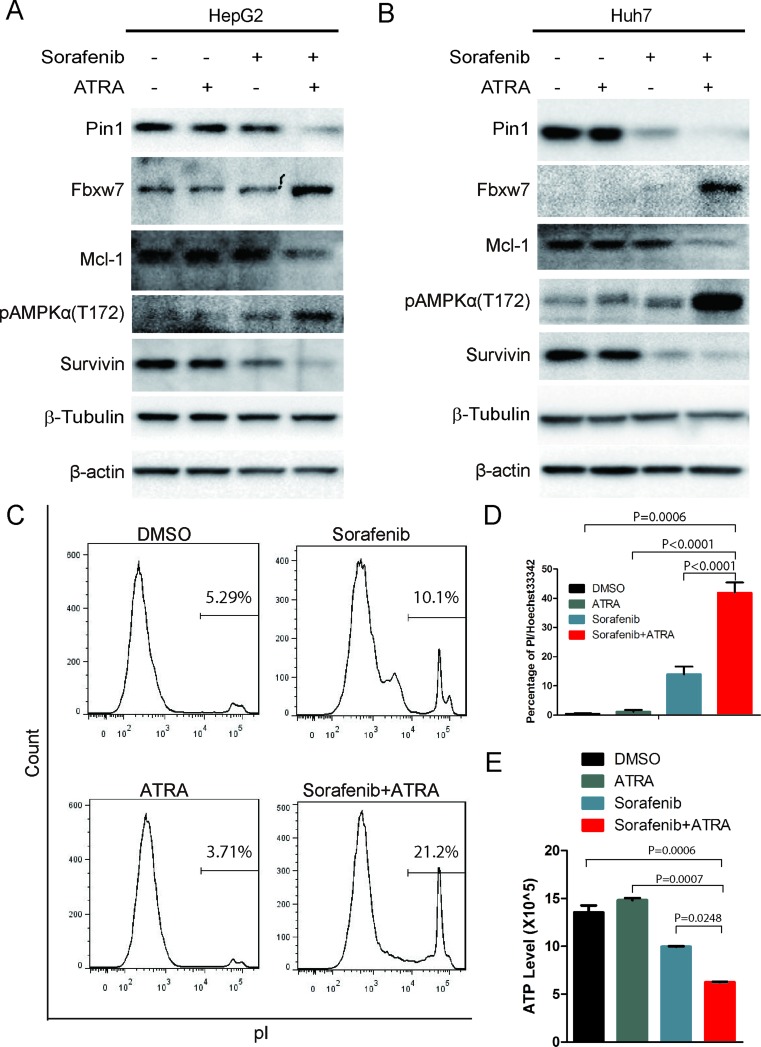
The Pin1 inhibitor ATRA sensitizes HCC cells to sorafenib-induced cell death.\ (**A**, **B**) ATRA synergistically enhanced sorafenib-induced down-regulation of Pin1 in HCC cell lines. HepG2 and Huh7 cells were treated with 5 μM sorafenib, 25 μM ATRA or their combination for 24 hours; Pin1, Fbxw7, Mcl-1, survivin and tubulin protein expression and phosphorylation of AMPK on Thr172 were determined by Western Blot. (**C**, **D**, **E**) ATRA synergistically enhanced sorafenib induced cell death. Huh7 cells were treated with 5 μM sorafenib, 25 μM ATRA or their combination for 72 hours; cell death was stained by pI and analyzed with flow cytometry (C). Alternatively, cells were stained with pI and Hoechst 33342. The number of red and blue spots were counted using Image pro plus 6. Sorafenib induced cell death were determined by the ratio of Red/Blue (D). Huh7 cells were treated with 5 μM sorafenib, 25 μM ATRA or their combination for 36 hours. Intercellular ATP level was determined (E).

Since shRNA could have off-target effects and it is still very challenging to deliver shRNA to tumors for cancer therapy, we used a small molecular Pin1 inhibitor, ATRA, which has been identified through a mechanism-based screening from a compound library [41]. ATRA binds to active Pin1 selectively in cancer cells, leading to its degradation and exerting potent anti-tumor activity against acute promyelocytic leukemia and breast cancer [41]. Although 25 μM ATRA had moderate effects on Pin1 expression probably due to cytochrome P450-dependent ATRA metabolism in HCC cells, as we have shown [42, 43], ATRA significantly enhanced sorafenib-induced down-regulation of Pin1 expression (Figure 4A and 4B), demonstrating a synergistic effect of ATRA and sorafenib on reducing Pin1 expression. Accordingly, sorafenib and ATRA also had synergistic effects on Pin1 downstream targets, including expression of Fbxw7, Mcl-1 and survivin, and phosphorylation of AMPK on Thr172 (Figure 4A and 4B).

In order to investigate whether ATRA also enhances sorafenib-induced cell death, we treated HepG2 cells with increasing doses of sorafenib in the absence or presence of ATRA for 72 hours, followed by assaying dead and live cells using flow cytometry after pI staining, as described [44]. ATRA alone could not induce cell death, as shown previously [41] and sorafenib weakly induced cell death (Figure 4C), as shown before [13]. However, a combination of ATRA with sorafenib led to a significant increase in cell death (Figure 4C and 4D), supported by the synergistic reduction of the intracellular ATP level (Figure 4E). ATRA also dramatically enhanced sorafenib-induced inhibition of cell growth (Supplementary Figure 1A) and colony formation (Supplementary Figure 1B and 1C) in Huh7 cells. Furthermore, these results indicate that ATRA enhances the ability of sorafenib to reduce Pin1 expression and to induce cell death.

**Figure 5:**
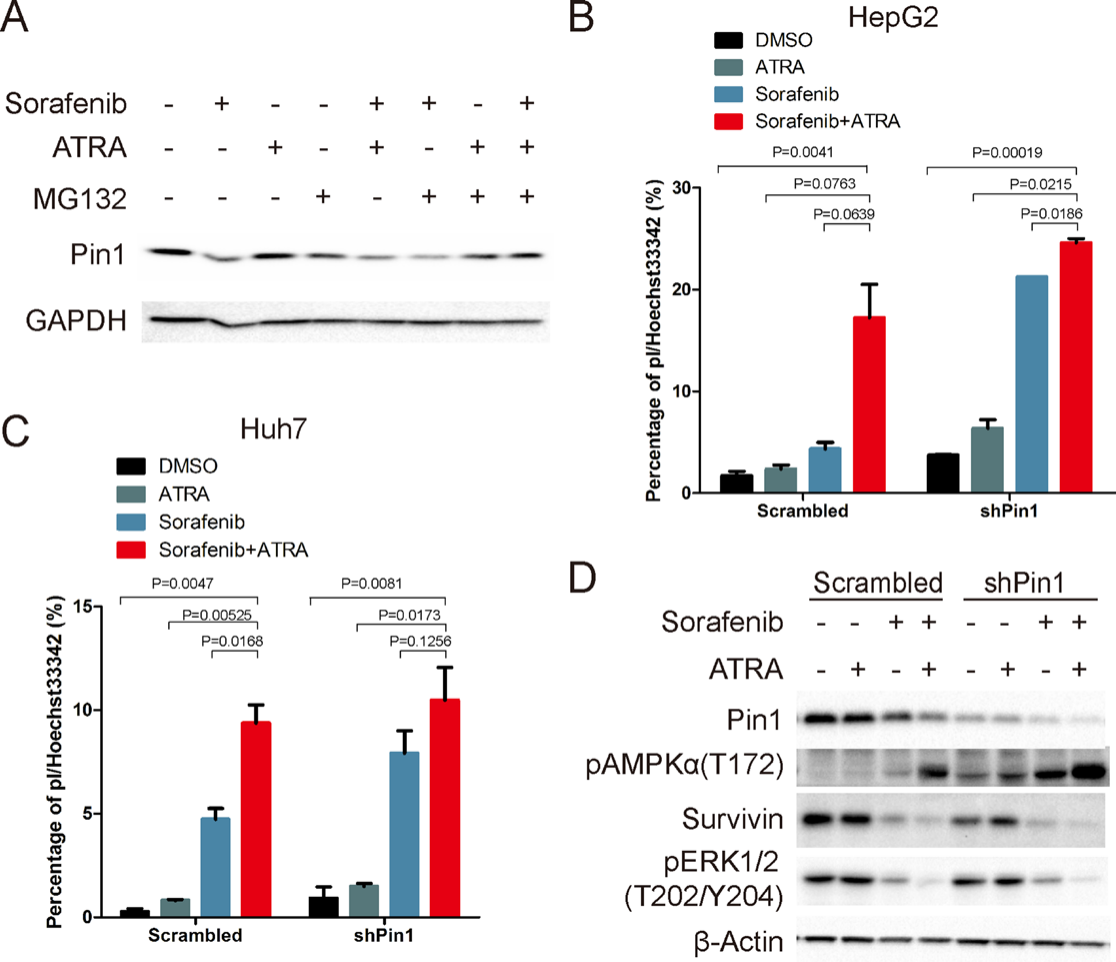
Sorafenib and ATRA synergistically induce cell death through down-regulating Pin1 (**A**) Cells were treated as indicated (sorafenib, 10 μM; ATRA, 25 μM; MG132, 10 μM) for 12 hours. Pin1 expression was determined by Western Blot. (**B**, **C**) HepG2 and Huh7 cells were treated as indicated (Sorafenib, 5 μM; ATRA, 25 μM) for 72 hours, stained with pI and Hoechst 33342, photographed and counted using Image pro plus 6. (**D**) Pin1 and survivin protein expression, and phosphorylation of AMPK on Thr172 and Erk1/2 on Thr202/Y204 were determined by Western Blot.

Since ATRA directly binds to Pin1, leading to inhibition of its enzyme activity and eventual its protein degradation [45], we proposed that the molecular mechanism underlying the synergistic Pin1 down-regulation induced by sorafenib and ATRA could be due to a combinational effect of sorafenib-mediated Pin1 biosynthesis inhibition and ATRA-mediated Pin1 degradation. Indeed, the proteasome inhibitor MG132 could partially reversed the synergistic effect, but not sorafenib-induced Pin1 down-regulation (Figure 5A). To investigate the role of Pin1 down-regulation in the combination of sorafenib- and ATRA-induced cell death, we compared the effects of sorafenib and ATRA in Pin1 knockdown and control cells. In both Huh7 and HepG2 cells, Pin1 knockdown attenuated ATRA-mediated enhancement of sorafenib-induced cell death (Figure 5B and 5C). Accordingly, Pin1 knockdown enhanced sorafenib-induced activation of AMPK and attenuated ATRA-mediated enhancement of sorafenib-induced activation of AMPK (Figure 5D).

**Figure 6:**
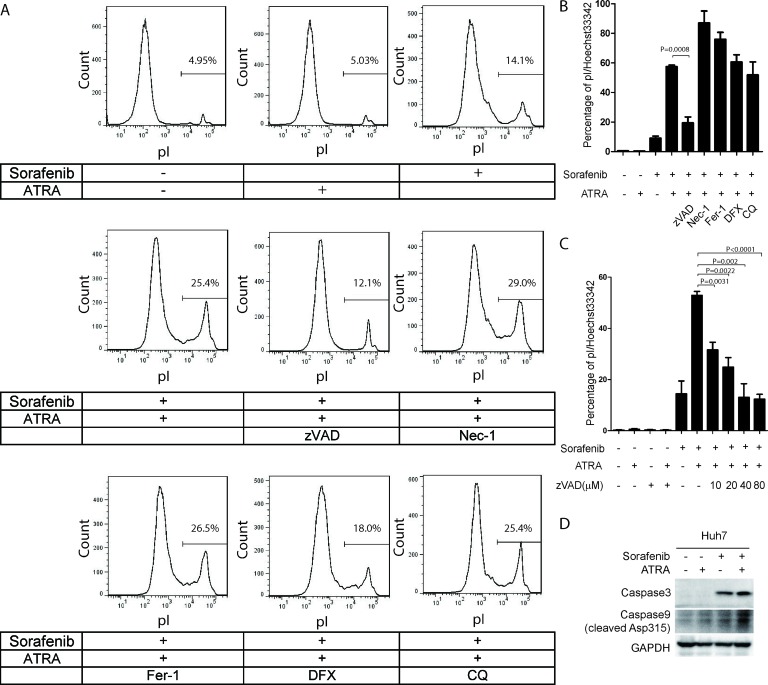
Cell death induced by the combination of sorafenib and ATRA is blocked by the caspase inhibitor zVAD in Huh7 cells (**A**, **B**) Cell death induced by sorafenib and ATRA was blocked by zVAD in Huh7 cells. Huh7 cells were treated as indicated (Sorafenib, 5 μM; ATRA, 25 μM; zVAD, 20 μM; Nec-1, 40 μM; Fer-1, 20 μM; DFX, 200 μM; CQ, 50 μM). Cell death was stained with pI and analyzed by FACs (A). Huh7 cells were treated as indicated, stained with pI and Hoechst 33342, photographed and counted using Image pro plus 6.0 (B). (**C**) zVAD blocked cell death induced by sorafenib and ATRA in a dose dependent manner. Huh7 cells were treated with different doses of zVAD, stained with pI and Hoechst 33342, photographed and counted using Image pro plus 6. (**D**) Huh7 cells were treated with 25 μM ATRA or 5 μM sorafenib or their combination for 48 hours. The protein levels of cleaved caspase 9 and caspase 3 were determined.

In order to determine the molecular mechanisms underlying cell death induced by the combination of sorafenib and ATRA, we treated Huh7 cells with several potent cell death inhibitors. Interestingly, zVAD, a caspase inhibitor, dramatically blocked cell death induced by the combination of sorafenib and ATRA (Figure 6A and 6B). In contrast, necrostatin-1 (Nec-1, a necroptosis inhibitor), ferrostatin-1 (Fer-1, a ferroptosis inhibitor), deferoxamine (DFX, an iron chelator) or chloroquine (CQ, an autophagy inhibitor) had little influence on sorafenib induced cell death (Figure 6A and 6B). Moreover, the effect of zVAD on cell death induced by sorafenib and ATRA was dose-dependent (Figure 6C). Supportively, synergistic activation of caspase 9 and caspase 3 were also observed under sorafenib and ATRA combinational treatment (Figure 6D). Together, these data indicates that ATRA enhances the ability of sorafenib to induce apoptosis in HCC cells.

### The Pin1 chemical inhibitor ATRA synergistically enhances the ability of sorafenib to inhibit tumor growth of HCC *in vivo*

**Figure 7:**
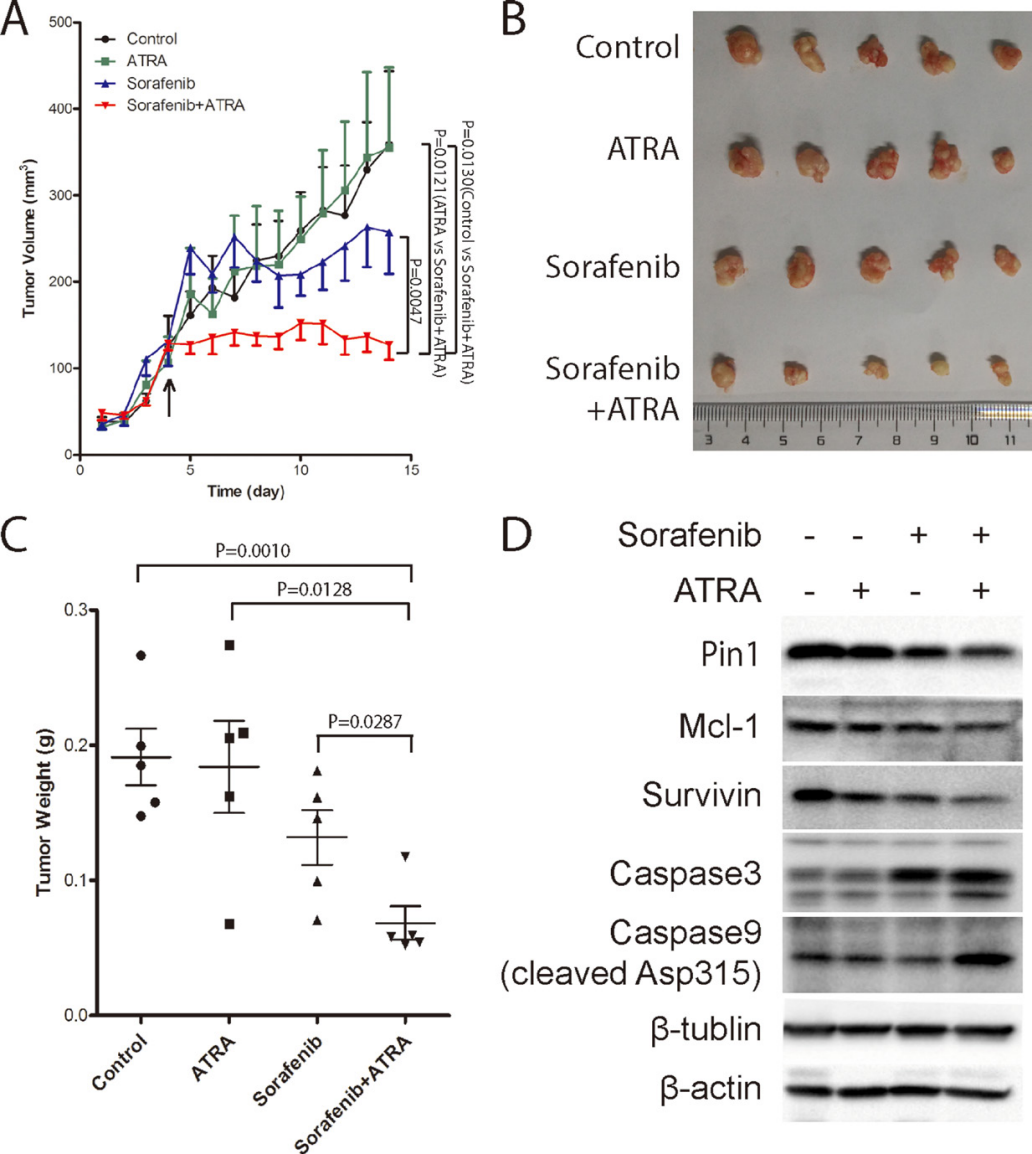
The Pin1 inhibitor ATRA synergistically enhances the ability of sorafenib to inhibit tumor growth of HCC *in vivo* (**A**) Tumor growth was significantly inhibited by the combination of sorafenib and ATRA. Huh7 cells were injected subcutaneously into nude mice. Drugs were administrated when tumors at the point indicated by the arrow. 1/4 tablet of 10 mg 21 day slow-releasing ATRA pellet was inoculated subcutaneously. Sorafenib (40 mg/kg) was orally given every three days. Tumor growth was measured every 3 days, and tumor volume was calculated using the formula of length*width*width/2. (**B**, **C**) ATRA synergistically enhanced sorafenib anti-tumor effect *in vivo*. Tumors were harvested (B) and weighted (C) when the length of largest tumor reached 1.0 cm. (**D**) ATRA synergistically enhanced sorafenib induced Pin1 down-regulation *in vivo*. Pin1, Mcl-1, survivin, beta-actin, beta-tubulin, cleaved caspase 9 and caspase 3, protein expressions were determined by Western Blot.

Given the potent ability of the Pin1 chemical inhibitor ATRA to enhance sorafenib-induced cell death in HCC cell lines, a critical question is whether ATRA would affect the anti-tumor efficacy of sorafenib in HCC *in vivo*. To address this question, Huh7 cells were injected subcutaneously into nude mice to establish xenograft tumor and 16 days later, when the tumors were detectable, mice were randomly grouped into four groups and treated with sorafenib, ATRA or combination of sorafenib and ATRA or vehicle saline, respectively. ATRA was implanted under the skin in the back of the neck using a slow releasing formula, as described [41]. Sorafenib was given by oral gavage, as described [10]. A low dose of ATRA (one-fourth pellet of 10 mg over 21 days) alone had the modest effect on tumor growth, while sorafenib alone did not inhibit tumor growth until the late stages (Figure 7A). By contrast, the combination of ATRA with sorafenib completely stopped HCC tumor growth, even leading to tumor shrinkage at the late stage (Figure 7A). When the tumors were harvested, weights of tumors treated with the combination of ATRA and sorafenib were only one-third of those treated with vehicle or ATRA alone, and one half of those treated with sorafenib alone (Figure 7B and 7C), demonstrating the strong synergistic effect of ATRA with sorafenib in inhibiting tumor growth *in vivo*. These results were further confirmed by the findings that Pin1, and its substrate effectors Mcl-1 and survivin, were down-regulated by sorafenib, and largely depleted by a combination of sorafenib and ATRA in tumors (Figure 7D). Consistent with the findings that a synergistic effect of sorafenib and ATRA on cell death could be blocked by the caspase inhibitor zVAD, cleavage of caspase 9 and caspase 3 were robustly detected upon combinational treatment of sorafenib and ATRA, indicating that sorafenib and ATRA synergistically inhibit tumor growth by inducing apoptosis. Taken together, these data consistently demonstrate that ATRA synergistically enhances the ability of sorafenib to induce Pin1 down-regulation, cell death and inhibit tumor growth of HCC *in vivo*.

## DISCUSSION

Sorafenib is the only medical treatment for the treatment of advanced HCC with a proven efficacy, but its effect is still limited [3–7]. Therefore, the identification of key factors and developing combinational drugs to enhance the efficacy of sorafenib in HCC is one of the attractive strategies. Here we show that sorafenib down-regulated Pin1 protein by inhibiting its biosynthesis. Genetic knockdown of Pin1 sensitized HCC cells to sorafenib-induced cell death. Moreover, the Pin1 chemical inhibitor ATRA not only enhanced the ability of sorafenib to induce cell death of multiple human HCC cells *in vitro*, but also synergistically potentiated sorafenib to suppress human HCC tumor growth in mice. These results demonstrate for the first time that Pin1 down-regulation is one of the key events underlying the anti-tumor effects of sorafenib and more importantly, uncover that Pin1 inhibitors offer a novel approach to enhance the therapeutic efficacy of sorafenib against HCC.

Sorafenib was originally designed to target on Raf family, which is a Ser/Thr kinase and a pivotal regulator of cellular proliferation [46]. Recently, an increasing body of evidence has shown that sorafenib has a profound impact on cellular signaling [9]. For example, Sorafenib turns off transcriptional and translational machinery by activating Rb and suppressing mTORC1, respectively [32]. In this study, we show for the first time sorafenib could down-regulate Pin1 expression at least in part through inhibiting its mRNA transcription by the E2F/Rb pathway. Given the fact that Pin1 promotes cancer development through activating more than 40 oncogenes/growth-promoting proteins and inactivating over 20 tumor suppressors [21], sorafenib induced Pin1 down-regulation might in part account for its multiple targeting effect in HCC.

It has become evident that blocking a single pathway may not be effective in solid tumors, especially aggressive or drug-resistant ones due to feedback and simultaneous activation of a wide range of interactive and/or redundant pathways [21, 47, 48]. Especially, liver cancer is a highly inter- and intra-tumor heterogeneous disease [49] so that targeting multiple cancer-driving pathways has its advantage in cancer therapy. Notably, Pin1 promotes cancer development by turning on and off dozens of oncogenes and tumor suppressors, respectively [21]. Moreover, Pin1 knockout mice develop normally [50] but are highly resistance to Ras, Neu/HER2 induced breast cancer [51] or Myc-induced Burkitt’s lymphoma [52]. These results indicate that Pin1 is dispensable in normal cells but is required for tumor cells, which confers Pin1 as an ideal anti-tumor candidate. These results implicate a promise for Pin1-targeting therapy in HCC, especially given its overexpression in about 70% HBV-related HCC [22]. However, to test this possibility was challenging due to lack of functional active Pin1 inhibitors until the recent identification of ATRA as a Pin1 inhibitor [21, 41].

The ability of ATRA to enhance sorafenib-induced cell death has been shown in acute myeloid leukemia [53] and liver cancer [54, 55], but the underlying molecular mechanisms are still largely unknown. In this study, we show that sorafenib and ATRA have strong synergic effect on Pin1 down-regulation both *in vitro* and *in vivo*, indicating a pivotal role of Pin1 on sorafenib induced cell death. Indeed, both genetic knockdown and chemical inhibition using ATRA could dramatically increase sorafenib induced cell death. This is of particular interest since enhancing sorafenib-induced cell death in HCC is expected to improve clinical benefit from sorafenib. Our xenograft mice data have shown that sorafenib alone leads to tumor stasis only later stages, but not shrinkage of tumor size, as shown previously [56], which could be attributed to its inhibitory effect on cell growth, but not cell survival. By contrast, the combination of sorafenib with slow-releasing ATRA not only potently stops HCC tumor growth, but also leads to decline of tumor volumes. These results suggest that clinical combination of sorafenib and the Pin1 inhibitor ATRA might be a new strategy to improve therapeutic efficacy in HCC. However, ATRA has a short half-life of 45 min in humans [57] and regular ATRA has moderate but detectable efficacy against solid tumors in some clinical trials, new generations of supposedly much more potent retinoid derivatives to target RARs or RXRs show little efficacy [58–62], which is likely due to the failure of these retinoids to inhibit Pin1 [41]. Taken together, these results provide a rationale for developing longer half-life ATRA or more potent and specific Pin1-targeted ATRA variants to overcome drug resistance in cancer therapy, especially in combination with sorafenib for HCC.

In summary, our results have identified Pin1 down-regulation as a key event underlying the anti-tumor effects of sorafenib, and also provide the strong rationale for further development of Pin1 inhibitors as a novel approach to enhance the therapeutic efficacy of sorafenib against HCC, one of the most lethal cancers.

## MATERIALS AND METHODS

### Cell culture and reagents

HCC cell lines HepG2, Huh7, Hep3B and PLC/PRF/5 cells were obtained from Cell Bank of Chinese Academy of Sciences. HepG2 and Huh7 cells were cultured in high glucose DMEM (#12800017; GIBCO), while Hep3B and PLC/PRF/5 cells were cultured in MEM (#SH30024.01; Hyclone). All medium were supplemented with 10% fetal bovine serum (#10437-028; GIBCO) and 100mg/mL streptomycin and 100U/mL penicillin (#SV30010; Hyclone). Cells were incubated in 37 degree with 5% CO_2_. Sorafenib were purchased from Santa Cruz Biotechnology (#sc-220125, Santa Cruz, CA) and Medchemexpress (#HY-10201, Shanghai, China). Antibodies were obtained from the following sources. Pin1 antibodies were previously described [63]. GAPDH (#HC301-02), beta-actin (#HC201-02), beta-tubulin (#HC101-02) were from Transgen (Beijing, China). Fbwx7(#55290-1-AP), Mcl-1 (#16225-1-AP) and survivin (#10508-1-AP) antibodies were purchased from Proteintech (Wuhan, China). Bax (#5023S), Phospho-Rb(Ser807/811) (#D20B12) and phospho-AMPKa(Thr172) (#2535P) antibodies were from Cell Signaling Technology (Danvers, MA). Caspase 3 (#PA5-23921) and Cleaved caspase 9 (#PA5-17913) antibodies were from ThermoFisher Scientific (Rockford, IL). ATRA (#R2625), MG132 (#C2211), propidium iodide (#P4170) and Hoechst33342 (B2261) were obtained from Sigma (St. Louis, MO). Cell counting Kt-8 (CCK-8) was from were from Transgen (Beijing, China). CellTiter-Glo^®^ Luminescent Cell Via- bility Assay Kit was from Promega (2800 Woods Hollow Road, Madison, WI, USA). ATRA slow releasing pellet (#V-111) was purchased from Innovative Research of America (Sarasota, FL).

### RNA interference

Pin1 shRNA (5′-CCACCGTCACACAGTATTTAT-3′) was previously described [34]. To establish stable Pin1 knockdown cell lines, Huh7 and HepG2 cells were infected with lentiviruses expressing Pin1 shRNA. Stably knockdown cells were selected with 0.5 mg/mL puromycin.

### Cell death assay

pI/Hoechst double staining followed by microscopy was performed as previously described [35, 36]. Briefly, HCC cells were treated as indicated and stained with 5 ug/mL pI and 5ug/mL Hoechst 33342, examined under fluorescence microscopy (Zeiss Axio Observer A1). pI positive cells were considered dead or late apoptotic cells, whereas Hoechst 33342 positive cells were blue and considered normal or early apoptotic cells. pI or Hoechst33342 positive cells were counted using Image pro plus 6.0 (Media Cybernetics, MD, USA), respectively.

pI staining followed by flow cytometry was conducted as previously described [44]. Briefly, HCC cells were treated as indicated and stained with pI alone. pI incorporation and cell size were quantified by flow cytometry. pI negative cells with normal size were considered as live cells. pI positive cells with smaller size were considered as dead cells.

### ATP assay

Equal volume of reagents from CellTiter-Glo Luminescent Cell Viability Assay Kit was directly added to media. Record luminescence at Microplate Luminometer (Orion-L2) from BERTHORD, Germany.

### CCK8 assay

10 μL of CCK8 was added into 90 ml of media. Cells were then incubated at 37°C and 5% CO2 for one hour. Measure the absorbance using Multiscan GO from Thermo Scientific at 460 nm. The reference wavelength was 600 nm.

### Animal models

All animal protocols were approved by Experimental Animal Ethics Committee of Fujian Medical University. BALB/c nude mice were maintained in specific-pathogen-free Laboratory Animal Center of Fujian Medical University.

For tumor implantation, 2.5*10^6^ Huh7 cells were inoculated subcutaneously onto the left and right flank region of ~6 week old nude mice. Tumor volume was measured every three days and calculated by the formula: Length*width*width/2.

For ATRA treatment, one-fourth pellet of 10 mg ATRA with slowing releasing formula for 21 days was embedded under the neck skin. For sorafenib treatment, 40 mg/kg sorafenib was given every three days by oral gavage.

### Statistical analysis

Experiments were routinely repeated at least three times. All data are presented as the means ± SD, followed by determining significant differences using the two tailed student *t* test.

## SUPPLEMENTARY MATERIALS FIGURES AND TABLES


