# Supplementary material for: Inhibition of the prolyl isomerase Pin1 enhances the ability of sorafenib to induce cell death and inhibit tumor growth in hepatocellular carcinoma
Source: Oncotarget. 2017 Mar 7;8(18):29771–84. doi: 10.18632/oncotarget.15967 (PMC5444702; doi:10.18632/oncotarget.15967)
Supplement: Supplementary file 1 [file oncotarget-08-29771-s001.pdf]

Inhibition of the prolyl isomerase Pin1 enhances the ability of sorafenib to induce cell death and inhibit tumor growth in hepatocellular carcinoma

Supplementary Materials

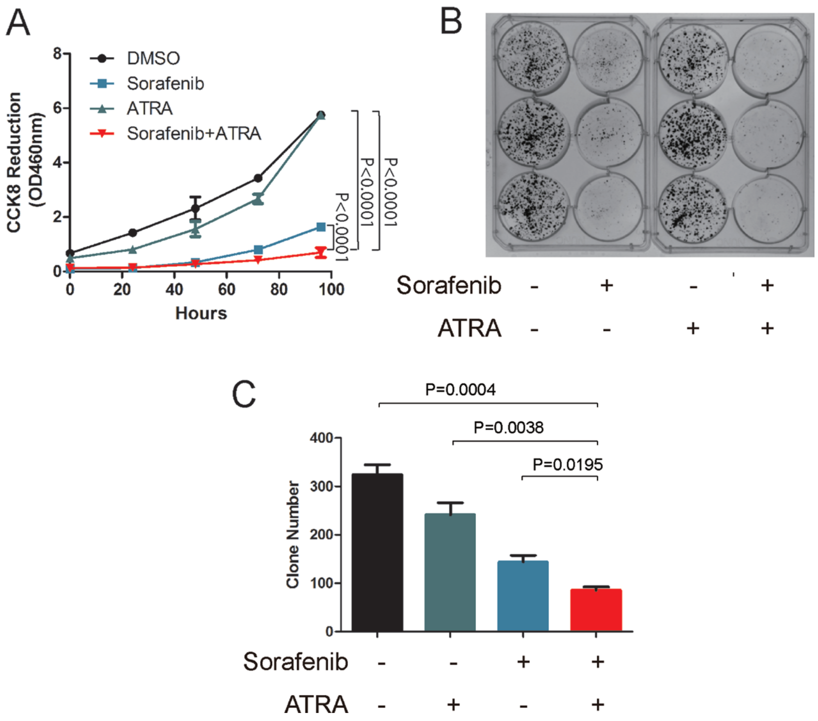

Supplementary Figure 1: Sorafenib and ATRA synergistically suppress cell grow and colony formation in Huh7 cells. Huh7 cells were treated as indicated (Sorafenib, 5 µM; ATRA, 25 µM) for 48 hours, media were re-freshed with DMEM containing 10% serum. (**A**) Cell growth was measured by CCK8 assay per 24 hours. (**B**) Clone forming assay was performed. (**C**) Clone number was calculated using Image pro plus 6.
